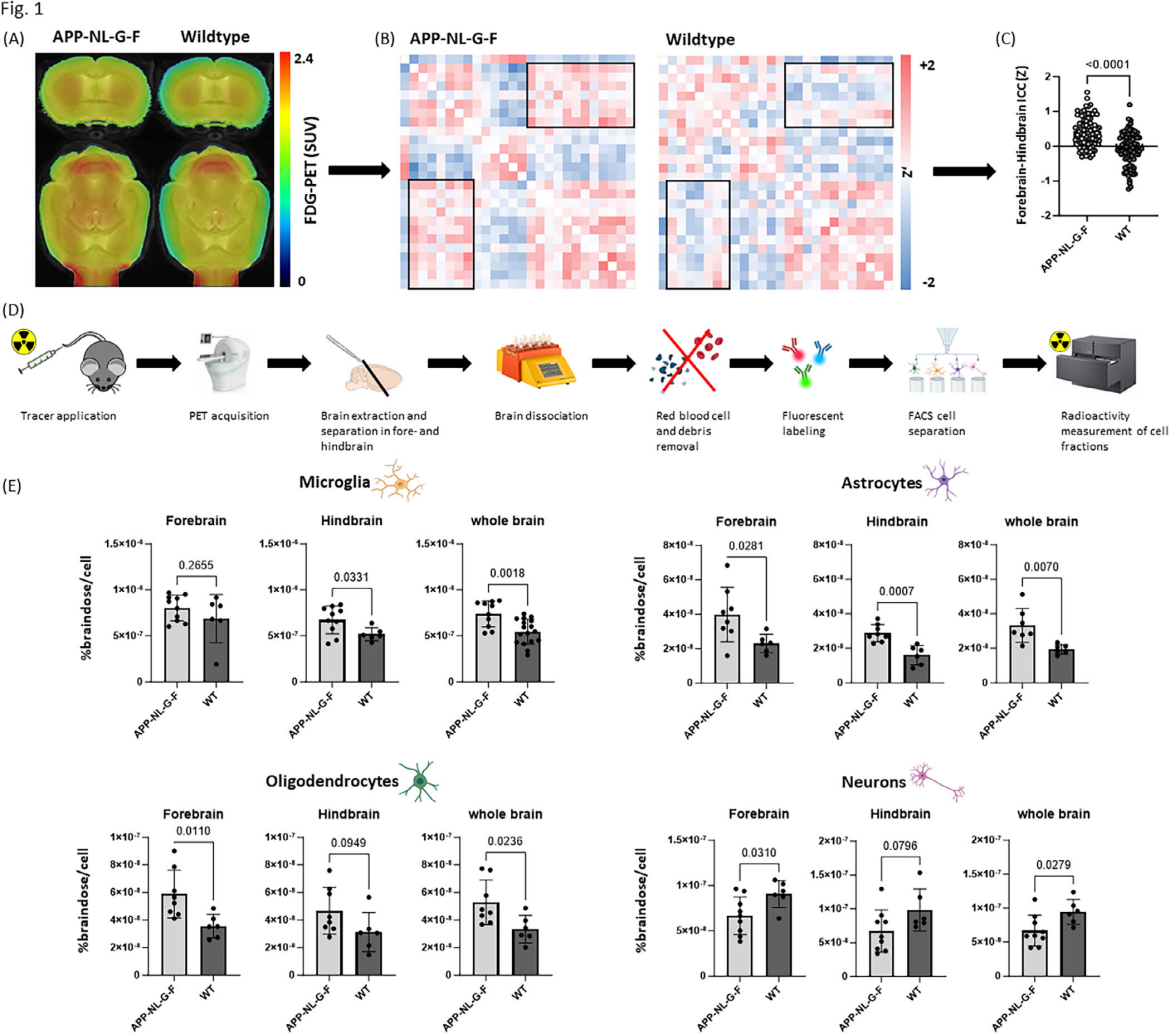# Cell‐Type Specific Contributions to Metabolic Connectivity in an Alzheimer's Disease Mouse Model

**DOI:** 10.1002/alz70855_105605

**Published:** 2025-12-24

**Authors:** Manvir Lalia, Stephan Wagner, Selina Hummel, Justus Thevis, Danilo Prtvar, Artem Zatcepin, Valerio Zenatti, Laura Bartos, Sabina Tahirovic, Matthias Brendel, Johannes Gnörich

**Affiliations:** ^1^ LMU University Hospital, Munich, Germany; ^2^ German Center for Neurodegenerative Diseases (DZNE), Munich, Germany; ^3^ Munich Cluster for Systems Neurology (SyNergy), Munich, Bavaria, Germany

## Abstract

**Background:**

The integration of molecular imaging and multivariate connectivity approaches has emerged as a novel approach to gain insights into the underlying pathophysiology in neurodegenerative diseases. Metabolic connectivity, in particular, has already demonstrated disease‐related pattern changes in both human and mammalian brains. However, the cellular sources of disconnected brain regions have not been investigated in detail. This study aimed to elucidate the driving cellular sources of metabolic connectivity in an Alzheimer's disease (AD) mouse model and wild‐type mice (WT).

**Method:**

After intravenous injection of 45MBq F‐18‐FDG, a static PET/MRI was performed on APP^NL‐G‐F^ and age‐ and sex‐matched WT controls to obtain maps of regional FDG uptake and metabolic connectivity. To calculate the inter‐regional correlations for metabolic connectivity, 26 delineated brain regions were used, resulting in a 26 × 26 matrix of Pearson's correlation coefficient pairs. Subsequently, the brain was extracted and separated into fore‐ and hindbrain to achieve region‐specific isolation of microglia, astrocytes, oligodendrocytes, and neurons. The radioactivity of each cell fraction was measured to quantify the cell‐specific FDG‐uptake (Figure 1D).

**Result:**

APP^NL‐G‐F^ mice demonstrated higher FDG uptake compared to WT, along with a significantly increased metabolic connectivity between fore‐ and hindbrain (Figure 1A‐C). Among all cell types, microglial exhibited the highest single‐cell FDG uptake, in both mouse models (Figure 1E). In APP^NL‐G‐F^ mice, microglia, astrocytes, and oligodendrocytes displayed increased FDG uptake, while neurons exhibited reduced uptake compared to WT. The correlation between forebrain and hindbrain cellular FDG uptake was significant across all cell types in the APP^NL‐G‐F^ model (microglia r=0.89, *p* = 0.0006; astrocytes r=0.65, *p* = 0.042; oligodendrocytes r=0.77, *p* = 0.025 and neurons r=0.51, *p* = 0.005). In contrast, WT mice did not exhibit any significant correlation in single‐cell uptake between forebrain and hindbrain. Notably, region‐specific microglial FDG uptake correlated significantly with respective FDG‐PET signals in APP^NL‐G‐F^ mice (forebrain r=0.89, *p* = 0.007; hindbrain r=0.8, *p* = 0.014), whereas no significant correlation was observed for other cell types.

**Conclusion:**

These findings suggest that microglia are the primary drivers of the increased forebrain‐hindbrain metabolic connectivity observed in the AD mouse model. Further RNA expression analyses could provide valuable insights into the molecular mechanisms underlying microglial metabolic coupling in neurodegeneration.